# Factors affecting acquisition of psychomotor clinical skills by student nurses and midwives in CHAM Nursing Colleges in Malawi: A qualitative exploratory study

**DOI:** 10.1186/s12912-016-0153-7

**Published:** 2016-05-04

**Authors:** Omero Gonekani Mwale, Roselyn Kalawa

**Affiliations:** Department of Community and Mental Health, The Catholic University of Malawi, Limbe, Malawi; Department of Clinical Nursing, St Joseph College of Nursing and Midwifery, Limbe, Malawi

**Keywords:** Clinical competences, Clinical skill, Clinical teaching, Skill acquisition in the clinical area, Nurse student

## Abstract

**Background:**

Acquisition of psychomotor clinical skills has been shown to improve the quality of care provided to patients when care providers are competent. The aim of this study was to explore students, nurses and tutors experience on factors affecting acquisition of psychomotor clinical skills.

**Methods:**

The study employed an exploratory qualitative research design. The population was students, clinical nurses and tutors from a nursing College and mission hospital in the southern region of Malawi. In depth interviews using a semi structured guide was used to collect data. Thematic analysis method was employed to analyze the collected data. Ethical principles of respect of human dignity, beneficence and justice were observed.

**Results:**

The findings have shown that acquisition of psychomotor skills is affected by: student motivation, lack of resources, learning environment, knowledge gap between the qualified nurses and tutors, and role modeling.

**Conclusion:**

In principle when student nurses have acquired necessary skills the quality of care provided to patients improve. Basing on the findings of this study it is recommended that Student should be well prepared before clinical placement Nurses and tutors should also update their knowledge and clinical teaching skills for them to adequately guide students. The clinical arena should have adequate resources.

**Electronic supplementary material:**

The online version of this article (doi:10.1186/s12912-016-0153-7) contains supplementary material, which is available to authorized users.

## Background

The ability to provide quality health care depends on the philosophical assumptions and the methods that have been installed in aspirant student nurses during their training. Since all health care systems are labour intensive, they require properly trained, well qualified and experienced nurse practitioners to produce optimal results [1]. Since those who train student nurses are the ultimate custodians of the standards and quality of the nursing profession, they also function as the mentors of those whom they teach. The acquisition of the skills required for successful practice and learning can only be accomplished when student nurses are supported and guided by their superiors in their learning environments.

The acquisition of psychomotor clinical skills is an essential part of learning to be a nurse/midwife and a lack of clinical skills competency can compromise patient care and safety [2]. When student nurse/midwives are well trained in clinical skills, the quality of care for patients in the hospitals and clients in the communities is improved. This training can well be done at the pre service level as it is stipulated in the curriculum for the CHAM Nursing Colleges. Students are supposed to start acquiring clinical skills during theory and practice in the skills laboratory. This skill acquisition continues in the clinical area. The role of the clinical instructor in this case is to help the students acquire necessary skills, which have direct effect on patient care as they are the benchmark for nursing and midwifery practice. This is stipulated in the curriculum which is being implemented in all CHAM Nursing colleges in Malawi.

The recommended curriculum contains the following courses that are taught to students at different levels. In level one students are taught introduction to psychology, sociology, pharmacology, parasitology & microbiology, professionalism,introduction to fundamentals of nursing, introduction to medical/surgical nursing chemistry and physics, language and communication.

While at level two, students are taught human nutrition, community health nursing, medical/surgical nursing, mental health & psychiatric nursing. In third year, students are taught midwifery science I, midwifery science II and neonatology. When students have learnt theory, they are allowed to practice in the skills laboratory to master the skills before they are exposed to real clients in the clinical area.

Quality nursing care is compromised due to shortage of well-trained nurses. Most of the nursing care is provided by Nurse Midwife Technicians (NMTs) in Malawi.

High quality health care demands a nursing workforce with competence in the clinical skills. However the clinical competency of newly qualified nurses from CHAM Nursing Colleges continues to stimulate debate about the adequacy of current method of clinical skills education. Such that, people have wondered as to how nurses and midwives are being trained in CHAM Nursing Colleges. Despite the curriculum and improved skills laboratories in most CHAM Nursing Colleges, still nurses and midwives graduating from these colleges are not competent enough to deliver the quality care to patients in most hospitals in Malawi. It has also been observed that, there is an outcry by the public with the decline of nursing standards in Malawi [3]. Of particular note, is lack of published research in Malawi on factors affecting acquisition of psychomotor skills in the clinical area by student nurses and midwives. It was against this background that this research study was conducted with the purpose of exploring the factors that affect the acquisition of psychomotor clinical skills by students graduating from CHAM Nursing Colleges.

Specifically this study aimed at describing: (1) students experiences on the factors that hinder acquisition of psychomotor clinical skills and (2) describing nurses and tutors experiences on factors that hinder acquisition of psychomotor clinical skills.

The study was guided by the following research question. What are the factors that influence acquisition of psychomotor skills by student nurse/midwives?

### Definition of operation terms central to the research

In order to have the same understanding between the reader and researchers the following terms have been defined.

Clinical nurse: A Qualified person registered with nurses and midwives council of Malawi who provides care to patients
Tutor: A qualified nurse who teaches students in a one to one or small group interaction
Clinical supervisor: A qualified nurse solely employed to support students in the hospital.
Clinical teacher: A qualified person who teaches students in the clinical area during placement.
Student nurse/midwife: According to Nurse and Midwives Council of Malawi defines a student nurse/midwife as a person who is undergoing active learning or who is studying to enter a particular profession.
Clinical instructor: A qualified nurse whose responsibility is to provide instructions to students during clinical practice.

## Methods

### Study design and context

Descriptive and exploratory methods were used because little was known on the topic in Malawian nursing education. This design was ideal because qualitative methods are primarily concerned with in-depth study of human phenomena in order to gain insight and understanding of the phenomena [4].

The study was conducted at St Joseph College of Nursing and St Joseph hospital located in the southern region of Malawi. St Joseph College of Nursing is one of the nursing colleges which trains the nurse midwives technician. It enrolls 250 nursing students per year.

St Joseph hospital has a bed capacity of 200. It provides integrated health services to the marginalized population.

### Participants

The participants for this study therefore were students in second year, third year, tutors and nurses working at the hospital.

The sample size of the participants was influenced by the nature of the design and the number of respondents. Eleven students were recruited into the study of which 7 were from year two and 4 were from year three. Similarly, 2 tutors, with 1 and 6 years work experience represented the sample. Two clinical nurses with 5 and 7 years work experiences also represented the sample from the clinical site.

### Sampling

Generally, a purposive sample is comprised of respondents who are likely to be able to provide information and the phenomenon under study [5]. In this case, tutors and clinical nurses were targeted as a sample who had at least 1 to 7 years work experience.

The identified participants were then approached by the researcher and requested to participate in the study. Written consent was mandatory for enrollment for the study.

The sample size for qualitative research was not predetermined and therefore sampling was done until saturation, when no new data emerged, but previously collected data were repeatedly reintroduced [6, 7], states that data saturation has “become the gold standard by which purposive sample sizes are determined in health science research” and they suggest that data saturation can occur after 12 interviews. According to these authors, smaller sample sizes can be sufficient in providing complete and accurate information within a particular cultural context, as long as participants posses a certain degree of expertise about the domain of inquiry. It is argued that these experiences contribute to the participants’ sense of reality and “truth” [7]. Therefore, the researcher recruited 15 participants in this study.

### Data collection

Data was collected through in-depth interviews using a semi structured interview guide (see Additional file 1). This method was used because it provided a basis for convergence on truth because it was hoped that true information could be sorted out [8], state that semi structured interview guide provides a researcher with an opportunity to probe more on the issues under study.

An audio tape recorder with permission from the participant was used so that no data was missed. At the end of each interview, participants were asked if they wished to make any comments pertaining to the study topic. Following this the researcher thanked each participant for their time and valuable contribution. The interview took approximately 45 min with each student and 30 min with the tutor and clinical staff nurses respectively.

### Data analysis

Thematic analytic steps of Aronson 1994 cited in [9] guided analysis of factors affecting acquisition of psychomotor skills by student nurses in CHAM colleges in Malawi. The transcribed data and written file notes made by the researcher following each interview provided the means to begin exploring the data obtained. These reflections resulted in refinements to the interview schedule during the first two students’ interviews. Each interview was conducted by the researcher and then transcribed. This approach of re-reading the transcript allowed the researcher to view the preliminary data and highlight any initial ideas as they became evident. Importantly, it also enabled the exploration of other issues that may not have initially been considered central to the research question [10]. The transcribed interviews were read and re-read to allow the researchers to familiarize with the data. This strategy meets Lincoln and Guba’s 1985 as cited in [11] requirement of data immersion being an inductive rather than deductive activity that requires repeated exposure to and engagement with the data. Immersion in the data enabled the researcher to see how the participants’ perceived factors affecting acquisition of psychomotor skills by student nurses. It also allowed the researchers to fully comprehend how insights were grounded in and developed from the data with the emphasis on understanding participants’ experiences through their descriptions [12]. The researchers read each transcript as a whole while listening to the audiotape to gain a sense of the participant’s entire story and to reflect on comments, phrases and any associated vocal qualities that stood out. Patterns and themes that emerged from the data were noted and highlighted in different colours and ideas and thoughts were noted next to particular parts of the text in order to clearly track the researcher’s emerging observations about the data, rather than searching for instances that reflected a previous theoretical position (Lincoln & Guba 1985 cited in [11, 12]). Key terms or phrases together with the corresponding text that illustrated the key terms and phrases were highlighted and then assigned a code [9]. Transcripts and codes were discussed by the research team and reviewed accordingly. The results of the preliminary coding by the researcher and co-researcher were then compared and any differences discussed until consensus was reached. Coded concepts from each transcript were identified and Sub-themes derived from the codes. Two major themes emerged with eight (8) subthemes (See Table 1).

**Table 1:** Summary of data analysis

Themes	Sub-themes
The clinical environment	Student motivation
	Support and clinical supervision
	Shortage of material and shortage of nurses
Learning opportunities	Student allocation
	Role model
	Time for practicing in the clinical area
	Interpersonal relationship
	Knowledge gap

### Trustworthiness of the study

*Trustworthiness* is a measure of the extent to which a researcher’s findings reflect the truth about particular situations or entities. All research findings should (as far as is humanly possible) exclude bias and inaccuracies [13]. In the opinion of [14], trustworthiness refers to the quality, authenticity and truthfulness of the findings in qualitative research.

#### Strategies that the researcher applied to enhance trustworthiness

The researcher engaged the clinical facilitators in a discussion in such a way that they were motivated to give their inputs spontaneously and enthusiastically. This face to face interview was verified by means of triangulation, which is one of the strategies used to enhance the *trustworthiness* of any research. The clinical facilitators then proceeded to discuss the facts that constitute truth and the various means that one can use to minimize or eliminate bias [15]. The significance of this group was that it allowed the researcher to collect data from experienced and professional practitioners who were very different in their outlook because of their experience, knowledge and wisdom from the student nurses who constituted the first interviews. The clinical facilitators discussed the various ways in which they practiced the clinical supervision of the student nurses in clinical settings in the hospital and they also provided input into the formulation of guidelines that might be useful for professional nurse educators who were charged with responsibility for the clinical supervision of student nurses in the future.

## Results

The major themes that emerged from the transcribed data were: The clinical environment with subthemes of Student motivation, lack of material resources and human resources. Learning opportunities in the clinical area with subthemes of student allocation, role modeling, time for practicing in the clinical area, interpersonal relationship and Knowledge gap between the tutors and clinical staff (See Table 1).

### Clinical environments

Under this theme three subthemes emerged from the transcribed data.

### Student motivation

One of the findings from this study on factors that affected skills acquisition was student motivation. It was mainly reported by clinical nurses and tutors that students are not motivated to learn the skills as they lack initiative in their own learning and one tutor said this.

*‘Individual interests of the students vary; some are not mature enough to handle situations”.*

The same tutor continued to say;

*‘Some students do not have personal interest they come to nursing because they are told to do so’.*

Another tutor pointed out that if students had to acquire the skills they needed to have interest in the profession since the supervision that is done is not enough, as such students should have the personal initiative to learn. In agreement to this, one nurse said.

*‘Other students are reluctant to be taught and they undermine the knowledge they are taught from the clinical nurses’.*

When students are well prepared they are motivated and the opposite is true that if not well prepared they are demotivated. One student said;

*‘we were given objectives and the tutor explained what we were to do in the clinical area’.*

While there were mixed views on this area it seemed that students had a strong feeling that it is the presence of patients that would enable them to acquire the skills. Another student said that it is the set up of the hospital that would motivate or demotivate someone to acquire the skills not necessarily the presence of clients alone. She went on to say that.

‘*The hospital set up, that is to say I was at a central hospital and there were a lot of patients on whom we could learn the skills on unlike the mission hospital’.*

There was also widespread agreement that preparation of students enhances the acquisition of psychomotor clinical skills in the clinical area.

### Support and supervision

The issue of competence guidance by tutors and clinical staff emerged prominent from many participants around acquisition of psychomotor clinical skills.

Many students said much on the provision of competence guidance by qualified nurses and tutors. It was uncommon to hear statements such as:

*‘Clinical staffs were assisting us a lot because there were some things that we did not cover in class but we managed to do those things with clinical nurses ‘Tutors were able to demonstrate the required skills in the clinical area’*.

### Shortage of materials

The findings further have shown that students were not able to acquire the skills due to lack of resources in the clinical area. This was reported by one student who said;

*‘Equipment was not available in the ward as a result there was a lot of improvision for example, there was no mackintosh in the ward as such the women were told to bring their plastic papers, some could not even afford and some were bringing dirty ones’.*

Furthermore, most students mentioned that despite the lack of equipment in the clinical area they were still compelled to work due to the presence of patients. One student stated this:

*‘The presence of clients compelled us to work on hence acquiring the skills, because with no clients there is no way we could have acquired the skills”.*

### Shortage of clinical nurses

Even though it was mentioned that students can acquire the psychomotor clinical skills easily when they are provided with competence guidance, on the other hand non availability or inadequacy of qualified nurses for the student in the clinical area, affected skill acquisition. One student reported.

*‘We were left alone in the labour ward without being supervised by neither the Tutor nor the clinical nurse’.*

### Learning opportunities

#### Role modeling

The findings of this study have also shown that role modeling for the clinical practice has an influence on skill acquisition by students. This was elaborated by one student who said;

‘*Qualified nurses were role models to me because they provided good care to the patients’.*

On the other note some students said that qualified staffs were uncooperative as they were saying it is not their duty to teach the students. One student reported this;

*‘One day I did not finish the procedure then my senior nurse shouted at me and as such I was afraid to ask him anymore.’*

It was further revealed that poor role modeling by nurses hindered skill acquisition. One student said.

*‘Mastering of the procedure was a problem as there were short cuts from qualified nurses.*’

And she continued to say,

*‘When giving medication we learnt that we should go to patient bed side and give the drug, but when we tried to do that clinical nurses were saying we were wasting time.’*

Similarly tutors shared the same view that clinical teachers are supposed to be good role models. It was learnt from one tutor, who strongly said,

‘*We tutors have to be exemplary so that students can emulate the way we perform the procedures’.*

#### Student allocation

Another factor that hindered acquisition of psychomotor clinical skills by students was: Too many students versus number of patients in the clinical area. It was reported by many students and this was summarized by one of them who said this:

*‘There was too much competition to find clients, for example, when you are allocated to a specific ward there are requirements for you to achieve, so if there are so many students in that area you tend to have problems on how to achieve your objectives’.*

#### Interpersonal relationship

The ability of clinical teachers to interact with students, both on one to one basis and as a clinical group is another important teacher behavior. However it was reported that there was poor interpersonal relationship with the students and qualified nurse, as a result skill acquisition was compromised. One student reported this:

*‘Qualified nurses were not cooperative, when you try to ask them they would say just do it the way you learnt’.*

Another student also noted that this was from individual nurses as other nurses were willing to assist in the clinical area.

#### Knowledge gap

In this study it was also found that knowledge gap between nurses and tutors hindered clinical skill acquisition by students. Most students reported that they had noted a discrepancy when it came to performing of procedures among the tutors themselves as well as the clinical nurses.

One student reported.

*‘There is knowledge gap between what we learn in class and what the nurses in the ward know, for example management of second stage of labour’.*

#### Time for practicing

The findings have further indicated that time was also another factor that hindered skill acquisition by students in the clinical area. To support this, one tutor reported.

*‘We need to give students more time to practice in the skills laboratory as well as in the clinical area if the students have to be competent’.*

Students too, shared the same view that they were not staying long in a particular clinical placement as a result it was difficult to master a skill.

## Discussion

According to the findings of this study student motivation was observed to be outstanding as far as clinical skill acquisition is concerned. According to Atkinson [16], motivation gives a drive to a change in behaviour by arousing, sustaining and directing it towards the successful attainment of a goal. In this study some students explained that they were motivated to attain the skills because they had knowledge which they gained from theory, and they wanted to achieve by practicing those skills. This finding is similar to a study done by [17], who found that background knowledge of the nursing profession from family members was what motivated the students to join the career as such it could be difficult for the student to acquire the skills if not interested in the profession. The authors also feel that if the teaching methods are monotonous students might also be demotivated. The lack of human and basic resources in the clinical setting has been pointed out in this study to have an influence on skill acquisition by students. When there are no basic resources in the clinical area the nurses as well as the students resort to improvision. This improvision disrupts the process of acquiring the clinical skills, because most of the ideal steps are missed. Missing procedure steps might result in the students who are incompetent and unsafe to practice [18]. Stated that, students learn a clinical skill better if they are guided on how to perform that clinical skill.

In this study it was indicated the learning environment was influencing acquisition of psychomotor skills by students. It was learnt that students were not given enough time to practice a skill in the clinical area nor in the skills laboratory [19] asserts that psychomotor skills are an important part of nursing, however unlike other forms of learning; skills require practice and repetition of a procedure in order to be learnt. Therefore enough time must be set aside for drilling practical skills, and for demonstrating their complexity. Since students who practice learning skills in laboratory adapt more readily to the clinical field.

Poor interpersonal relationship between qualified nurses and student emerged as an issue under learning environment. Clinical teaching is supported by a climate of mutual trust and respect. Tutors and clinical nurses must respect students as learners and trust their motivation and commitment to the profession they seek to enter. Similarly students must respect the faculty’s commitment to both nursing education and society. The students themselves should also trust faculty members who will treat them with fairness and to the extent that it is possible not to allow the students make mistakes that would harm patients. Before expecting students to trust and respect clinical nurses, the clinical nurses themselves need to demonstrate their respect for students. Setting up a climate of mutual trust and respect takes time and energy. Even so but in the long run this improves the transferability of competences to students effectively [20] asserts that, good interpersonal relationship between the students and qualified nurses is the beginning of whatever the student is going to be in the future. If the tutors or qualified nurses do not create a friendly atmosphere for students, it means that students will not learn well as they lack trust in the nurse. This is in line with [21], who states that clinical instructors may encounter difficulties with students such as personality conflicts and lack of respect on the part of the students to learn the skills. The study also uncovered that knowledge gap was influencing acquisition of psychomotor skills by students. This finding is supported by [22] who also found that the registered nurse were rating students lower and indicated that there was need for the students to be well prepared unlike the general nurses who felt that the students had enough knowledge and there was no need to assist them. To the authors understanding, they are agreeing to the findings since it requires a nurse who is knowledgeable about the subject matter in order to assist the students in the clinical area. This meant that nurses who do not have enough knowledge can almost not transmit necessary skills to the learners. This finding agrees with [23], who state that clinical teachers may be worried about their theoretical basis of their own practice. The question that often comes up is whether or not they are up to date with knowledge and their ability to teach and model clinical skills.

Similarly [22], in their study also found similar results whereby nurses who had low education level felt that student did not need to be assisted as they felt inferior unlike the nurses who were highly educated as they were able to identify the needs of students. In the same way, since the students are assisted by different cadres of different experience and educational level in various hospitals, this could be true as regards to the knowledge gap which is there among these carders that eventually affects the transmission of skills to students. Effective clinical teachers need to be prepared and keep updated with current trends in nursing and midwifery. This was supported by [24], who observed that students expected their clinical teachers to be knowledgeable and skilled in the fields of nursing and midwifery.

In addition [25], uncovered that one of the most important professional responsibilities of nurses is to keep up with ever changing standard of practice. Health care is always dynamic therefore nurses must seek continuing education.

The study also indicated that poor role modeling had an impact on acquisition of competences in skills by students in the clinical area. This is in line with the findings of [26], who found that in the nursing homes students were not able to acquire the necessary nursing fundamental skills because of poor role modeling. Nursing students must be competent and efficient whilst carrying out clinical skills. However this may be threatened when students observe different practices of qualified nurses. Often for qualified nurses the familiarity of carrying out the clinical skills and low staffing levels are reasons why skills are not conducted in accordance to the nursing standards [19]. However, as the students in this study will be the professional graduates of the future and responsible for teaching others, it is imperative that they are taught correct clinical procedures. This will ensure that in the future they provide high quality patient care and satisfactory level of peer education [27], argues clinical nurses and tutors need to continually enhance the quality of teaching in practice as well as the quality of the placement themselves. To enhance the quality of teaching in practice and the quality of placements nurse educators must spend time in practice placements ensuring clinical skills are carried out correctly. According to [28] the key elements for nurse educators working in practice placements are liaising with students and clinical nurses, teaching clinical skills and providing evidence based practice. While [29] holds a different view as regards to clinical skill acquisition and continued to say that for the student to acquire psychomotor clinical skills there is need for the supervisor to be experienced as experience facilitates acquisition of clinical skills that leads into development of expertise. This in turn provides evidence based practice that improve outcome in the future hence excellence in health care.

### Limitations and strengths

The study is limited by the fact that all of the participants were drawn from only one of the (CHAM) nursing colleges in Malawi.

This was inevitable because during the period in which this study was undertaken, all the students from the other colleges were either on leave or were working in clinical placement which were unreachable. It would have been possible to base this study on a much wider range of views if the student sample had included students enrolled at all designated CHAM nursing colleges in Malawi. A further limitation is that there was very little literature on the topic in Malawi nursing education. However being the first study in Malawi, conducted with a limited sample the researcher was able to get the required information from the experienced participants.

### Recommendations

#### Nursing research

Since this research was conducted at one CHAM nursing college, the researcher therefore recommends that further research be conducted in the other CHAM nursing colleges in Malawi. Additionally, since the main focus of this study has been on the behavior, feelings, attitudes and opinions of the student nurses themselves. It would be valuable for the purpose of obtaining a more complete and nuanced understanding of these phenomena by conducting additional research into the experience of the clinical accompanists.

#### Nursing practice

It is further recommended that nurse tutors increase the frequency of visits to the wards when students are in clinical placement, so that each student can have a chance of being supervised by them. All procedural and standards be displayed at ward level so that students can to easily understand these tools, hence promoting patients care.

#### Nursing education

Knowledge gap was identified as factor that influences acquisition of psychomotor skills. It is further recommended that all training hospitals should be periodically updated with the curriculum content through continuous professional development.

## Conclusion

In principle acquisition of psychomotor competence in skills is important as it helps to provide quality care to patients. Consequently, a lack of skills competency can compromise patient care and safety [22]. It is therefore important that students should have an initiative for their own learning but at the same time the clinical environment should be conducive to allow learners to practice. This is essential if both the teaching hospital and training colleges realize many of the ideals enshrined in the learning process of a student.

### Ethics and consent to participate

Ethical permission was given by the National Health sciences Research Committee (NHSRC) to allow the nurse educators to conduct this research [ref/NHSRC#1049].

Permission was also sought from the College Principal and the hospital Manager. Written informed consent was obtained from all participants. Participants were briefed about the objective and procedures of the study. They were also informed about their right to agree or refuse to participate and their right to withdraw from the study at any time even after they had signed the consent form. Further, participants were made clear that they were not to receive any remuneration for participating in the study. Special permission was obtained from participants on the use of audio recorder during interviews. All participants who agreed to take part in this study signed an informed consent. Participants were also informed that information they provided would be treated with strict confidentiality and would only be used for the research purposes [30].

### Consent to publish

Not applicable.

### Availability of data and materials

Data supporting the findings is contained within the manuscript.

## Additional file

Additional file 1: Interview guide. (DOC 23 kb)

## Abbreviations

AIDS: Acquired Immune Deficiency Syndrome
CHAM: Christian Health Association of Malawi
DHS: Demographic Health Survey
HIV: Human Immune Virus
MDG: Millennium Development Goals
MOH: Ministry Of Health
MT: Midwife Technician
NCA: Norwegian Church Aid
NMT: Nurse Midwife Technician
WHO: World Health Organization
