# Supplementary material for: Factors affecting acquisition of psychomotor clinical skills by student nurses and midwives in CHAM Nursing Colleges in Malawi: A qualitative exploratory study
Source: BMC Nurs. 2016 May 4;15:30. doi: 10.1186/s12912-016-0153-7 (PMC4855342; doi:10.1186/s12912-016-0153-7)
Supplement: Additional file 1: — Interview guide. (DOC 23 kb) [file 12912_2016_153_MOESM1_ESM.doc]

Interview guide

**Title: Factors Affecting Acquisition of Competence in Skills by Student in CHAM Nursing College.**

Instructions

This instrument has three sections. First part is about demographic data of the participant and the second part is about factors that hinder acquisition of skills by the students. And the second part is about factors that facilitate acquisition of practical skills by students

College Identity............................ Serial Number of participant.......................

1. Demographic details

. Sex of the participant Male or Female (circle the appropriate)

2. How old are you? 3. What is your level of study?

4. In which department were you allocated to during the last clinical placement?

5. What were the competences in skills that were most interesting to you in these departments?

6. Which ones did you acquire?

7. How do you acquire the competence in skill in general?

8. How important is skill acquisition to you as a student nurse/ Midwife in patient care?

9. What were the skills you find it easy to achieve?

10. What do you think were the difficulty skills for you to achieve at your level?

11. Why were the skills mentioned above difficult?

12. Were you prepared before you went for this clinical placement?

If yes (how) ---If no (why) -----------

13. How long was your clinical placement?

14. What facilitated the acquisition of the competences in the clinical allocation?

15. Did you have any challenges in acquisition of the competences in the clinical placement? (If yes)

16. What were these challenges that influenced the acquisition clinical in skills?

17. What could be some of the ways of improving the acquisition of practical skills by the students?
